# Eugenol alleviates renal ischemia-reperfusion injury induced-endoplasmic reticulum stress via activating Sestrin2

**DOI:** 10.1016/j.clinsp.2025.100627

**Published:** 2025-03-25

**Authors:** Jingwei Liu, Xujie Sun, Junfeng Liang, Shiqiang Song

**Affiliations:** Department of Urology, Qingdao Chengyang People's Hospital, Qingdao, Shandong Province, PR China

**Keywords:** Eugenol, Renal ischemia-reperfusion injury, Oxidative stress, Endoplasmic Reticulum Stress, Sestrin2

## Abstract

**Introduction:**

Renal Ischemia-Reperfusion Injury (RIRI) often arises due to heightened oxidative stress, rendering it a central focus of research. Sestrin2 plays a pivotal role in regulating oxidative stress; nevertheless, its impact on the renoprotective properties of Eugenol (EU) during RIRI warrants further investigation.

**Methods:**

Mice and TCMK-1 cells were categorically assigned into six groups: Sham/Control, Ischemia-Reperfusion (IR)/HR (Hypoxia-Reoxygenation), IR/HR+EU, Sham/Control+Sestrin2-KO, IR/HR+Sestrin2-KO, and IR/HR+EU+Sestrin2-KO. The effects of EU and the involvement of Sestrin2 in RIRI/HR were evaluated using Urea Nitrogen (BUN), Creatinine (Scr), Superoxide Dismutase (SOD), Glutathione (GSH), Catalase (CAT), and Malondialdehyde (MDA) assay kits; western blotting; cell viability assays; HE-staining; and Reactive Oxygen Species (ROS) detection.

**Results:**

Following RIRI/HR, a marked deterioration in kidney function and a significant surge in oxidative stress levels were observed. However, EU treatment ameliorated renal injury and inhibited oxidative stress. Additionally, EU upregulated Sestrin2 expression, and the renoprotective effects of EU were reversed upon Sestrin2 knockdown.

**Conclusion:**

The present study posits that EU effectively mitigates RIRI/HRI (Hypoxia-Reoxygenation Injury), and its mechanism of renal protection potentially involves the upregulation of Sestrin2, coupled with the inhibition of oxidative and Endoplasmic Reticulum Stress (ERS).

## Highlights

• Eugenol significantly lessens renal injury and oxidative stress in ischemia-reperfusion.
• Eugenol's protection is enhanced by increasing Sestrin2 expression, crucial for its benefits.
• Eugenol protects kidneys by modulating oxidative and endoplasmic reticulum stress via Sestrin2.

## Introduction

Acute Kidney Injury (AKI) manifests as a clinical syndrome marked by a profound acute decline in renal function.^1^ Its heightened prevalence, perilous nature, and unfavorable prognosis underscore its urgency.^1^ Severe AKI may culminate in acute renal failure, precipitating swift renal function deterioration. Characterized by diminished glomerular filtration rate, rapid elevation of serum creatinine and blood urea nitrogen levels, and perturbations in water, electrolyte, and acid-base equilibrium, severe AKI poses a formidable challenge.^1^^,^^2^ Current clinical options for AKI are constrained, with renal replacement therapy emerging as the primary intervention.

Renal Ischemia-Reperfusion Injury (RIRI) stands as a common etiological factor in AKI pathogenesis. Despite the prevalence of AKI, therapeutic avenues remain limited, with renal replacement modalities being the principal recourse. Regrettably, the pursuit of effective interventions persists, leaving AKI treatment reliant on renal replacement therapies.^3^ This restoration triggers a cascade of inflammatory reactions and excessive free radical generation, resulting in cellular metabolic dysfunction and severe structural damage. Inflammation and apoptosis stand as hallmark pathophysiological processes in RIRI.^4^ Following RIRI, there is a substantial upregulation of inflammatory mediators and cytokines contributing to AKI.^4^ Additionally, apoptosis emerges as the predominant mode of death for renal tubular epithelial cells during transient renal ischemia/hypoxia.^5^ Under hypoxic conditions, the apoptotic signaling pathway is activated, prompting the expression of numerous apoptosis-related genes. Renal tubular epithelial cell apoptosis can lead to structural atrophy of renal tissue, a reduction in the number of renal cells, and further exacerbation of renal injury.^5^ Crucially, mitigating inflammation and apoptosis in renal tubular epithelial cells is imperative for preventing and treating RIRI.

Eugenol (EU) is a pale yellow, viscous, oily liquid derived from clove or other plants containing eugenol aromatic oil.^6^ A prior study has elucidated the diverse pharmacological activities of EU, encompassing antipyretic, analgesic, anti-inflammatory, antioxidant, anesthetic, memory improvement, and neuroprotective effects.^6^ Choudhary et al.^7^ demonstrated EU's capacity to regulate oxygen free radicals' scavenging and lipid peroxidation, leading to increased Superoxide Dismutase (SOD) activity, reduced Malondialdehyde (MDA) production, diminished Lactate Dehydrogenase (LDH) release, and a notable enhancement in the survival rate, coupled with a reduction in apoptosis in damaged cardiomyocytes. Consequently, the present study aims to elucidate the protective mechanism of EU against RIRI in mice, seeking to establish a theoretical foundation for its clinical application.

Sestrin2, a recently identified protein, plays a pivotal role in limiting Reactive Oxygen Species (ROS) accumulation and maintaining energy balance.^8^^,^^9^ A previous study demonstrated that Sestrin2 acts protectively against Ischemia-Reperfusion (IR) by inducing autophagy and activating the AMPK signaling pathway.^10^ The potential of EU as a natural antioxidant to modulate endogenous Sestrin2 expression raises intriguing questions necessitating scholarly investigation into its regulatory efficacy. Furthermore, the role of Sestrin2 in RIRI and its association with Endoplasmic Reticulum Stress (ERS) remain unclear. The prior findings have revealed that EU can up-regulate Sestrin2 expression in RIRI mice. Therefore, this study aims to scrutinize the precise mechanism behind the EU's anti-RIRI effects and the role of Sestrin2 in the context of RIRI.

## Materials and methods

### *Animals*

C57BL/6 wild-type mice (male, weighing 15‒20 g) and C57BL/6 Sestrin2 gene knockout mice (Sestrin2-KO; male, weighing 15‒20 g) were procured from GemPharmatech Co. Ltd (Nanjing, China) and housed at the Animal Center of Chengyang People's Hospital in Qingdao, Shandong Province, China. The mice received standard food and water, residing in a controlled environment with a 12-hour light/dark cycle, maintaining optimal temperature and humidity conditions.

### *RIRI model*

Before surgery, mice underwent an 8-hour fasting period. An intraperitoneal injection of 50 mg/kg pentobarbital sodium-induced anesthesia. Limbs were immobilized, and the surgical area was disinfected. An abdominal incision exposed the renal pedicle, and bilateral renal arteries were clamped for 45 minutes. Hemostatic forceps were released during reperfusion, with kidney color changes noted. The Sham group underwent a similar procedure without renal pedicle clamping. Kidney and serum samples were collected 24 hours post-surgery. Euthanasia was administered via an intraperitoneal injection of 250 mg/kg pentobarbital sodium, and the cessation of breathing and heartbeat confirmed mouse death.

### *Treatment protocols in vivo*

Fifteen mice were randomly assigned to three groups: Sham, IR, IR+EU, and fifteen Sestrin2-KO mice were assigned to three groups: Sham+Sestrin2-KO, IR+Sestrin2-KO, and IR+EU+Sestrin2-KO. To examine the EU's impact on RIRI, various EU doses (Cat. n° E110640, Aladdin, China) were administered via gavage seven days before inducing ischemia-reperfusion. All animal studies adhered to the ARRIVE guidelines and were approved by the Ethics Committee of Qingdao Chengyang People's Hospital (Approval n° Q2021–04–015).

### *Cell culture*

TCMK-1 cells (mouse renal tubular epithelial cell line) were cultured in DMEM (Cat. n° 12,430,112; Gibco, USA) with 1 % penicillin-streptomycin solution (Cat. n° 15,140,122, Gibco, USA), and 10 % fetal bovine serum (Cat. n° 16,140,089; Gibco, USA), maintained at 37 °C in an incubator with 5 % CO_2_.

### *Hypoxia-Reoxygenation (HR) model*

After a 12-hour serum-free DMEM starvation, TCMK-1 cells underwent 8 hours of hypoxia induction in a constant-temperature tri-gas incubator with 95 % N_2_.

### *Treatment protocols in vitro*

To explore Sestrin2 and EU's impact, TCMK-1 cells were incubated with EU at reoxygenation onset. Additionally, TCMK-1 cells were transfected with control siRNA (si-con) or Sestrin2 siRNA (si-Sestrin2; GenePharma Co., Ltd., Shanghai, China) 48 hours before HR. Cells were systematically allocated into six groups: Control, HR, HR+EU, si-Sestrin2, HR+si-Sestrin2, and HR+EU +si-Sestrin2.

### *Cell viability assay*

Cell viability was assessed using the CCK-8 assay kit (Cat. n° C0038, Beyotime, China). TCMK-1 cells were seeded into 96-well plates (1 × 10^4 cells/well), cultured for 12 hours, exposed to EU for 4 hours, and absorbance readings taken at 450 nm using a microplate reader (PerkinElmer, USA).

### *Renal function analysis and histopathological analysis*

Renal function was assessed by measuring plasma Creatinine (Scr) and Urea Nitrogen (BUN) levels at the Department of Clinical Laboratories of Qingdao Chengyang People's Hospital. Kidney samples were paraffin-embedded, sliced into 4 μm sections, stained with hematoxylin and eosin, and assessed according to Paller's scores.^11^ Two independent observers evaluated histopathological data.

### *Western blot*

Proteins were extracted from kidney tissues or TCMK-1 cells, lysed in RIPA buffer with protease and phosphatase inhibitors, and quantified using the BCA kit (Cat n° A045–4–2; Nanjing Jiancheng Bioengineering Institute, China). Protein samples (50 µg) underwent electrophoresis, transferred onto a PVDF membrane, and probed with primary antibodies overnight at 4 °C. Membranes were washed, incubated with secondary antibodies, identified using an ECL system, and band intensities quantified using ImageJ software (version 1.51). Details of the antibodies used are shown in Table 1.

**Table 1:** List of primary antibodies and secondary antibodies in Western Blot.

	Species	Dilution	Resources
**Primary antibodies**			
Sestrin2	Mouse	1:1000	Proteintech. China. Cat n° 66,297–1-Ig
ATF4	Mouse	1:1000	Proteintech. China. Cat n° 60,035–1-Ig
GRP78	Mouse	1:5000	**Proteintech. China.** Cat n° **66,574–1-Ig**
CHOP	Mouse	1:1000	**Proteintech. China.** Cat n° **66,741–1-Ig**
**Secondary antibody**			
HRP-conjugated Goat Anti-Mouse IgG		1:2000	**Proteintech. China. Cat n° SA00001–1**

### *The detection of oxidative stress indicators*

Levels of SOD, Glutathione (GSH), Catalase (CAT), and MDA in kidney tissues and TCMK-1 cells were determined using corresponding kits (Cat n° S0131S, S0101S, S0053; Beyotime, China) following the manufacturer's instructions.

### *The ROS levels of kidney tissues*

ROS content in kidney tissues was evaluated using a Dihydroethidium (DHE) fluorescent probe (D7008, Sigma-Aldrich, USA), as per the manufacturer's guidelines. Sections were incubated in 50 μM DHE for 1 hour, followed by staining with 1 mg/mL DAPI for 10 minutes at room temperature in the dark. Subsequently, sections underwent three washes with PBS. The fluorescence intensity was quantified using a fluorescence microscope with an excitation wavelength of 525 nm and an emission wavelength of 610 nm. The DHE-stained images were subjected to semi-quantitative analysis using ImageJ software (version 1.51).

### *The ROS levels of TCMK-1 cells*

To evaluate the levels of ROS in TCMK-1 cells, the authors utilized a DHE fluorescent probe according to the manufacturer's protocol. Following a 1-hour incubation with 25 μM DHE, the cells were stained with 1 μg/mL DAPI for 10 minutes in the dark. The samples were then subjected to three washes with PBS. Fluorescence intensity was measured using a fluorescence microscope, with an excitation wavelength set at 525 nm and an emission wavelength at 610 nm. The DHE-stained images were subjected to semi-quantitative analysis using ImageJ software (version 1.51).

### *Flow cytometry*

A total of 2 × 10^6 cells were subjected to two washes with PBS and then suspended in a staining buffer containing 1 µg/mL Propidium Iodide (PI) and 0.025 µg/mL Annexin V‑Fluorescein Isothiocyanate (FITC). The dual-labeling process occurred at room temperature for 10 minutes in darkness before initiating flow cytometric analysis. Subsequently, the cells were promptly analyzed using FACScan and the CellQuest Pro software (version 5.1; BD Biosciences, Franklin Lakes, NJ, USA).

### *Statistical analysis*

Data are presented as mean ± SD values and analyzed using GraphPad Prism software (version 8.0; GraphPad Software, Inc., San Diego, CA, United States) and SPSS software (version 26.0; SPSS Inc., Chicago, IL, United States). One-way ANOVA was used to analyze differences among multiple groups (*p* < 0.05).

## Results

### *Dose-dependent protective effects of EU on RIRI*

**Fig. 1:**
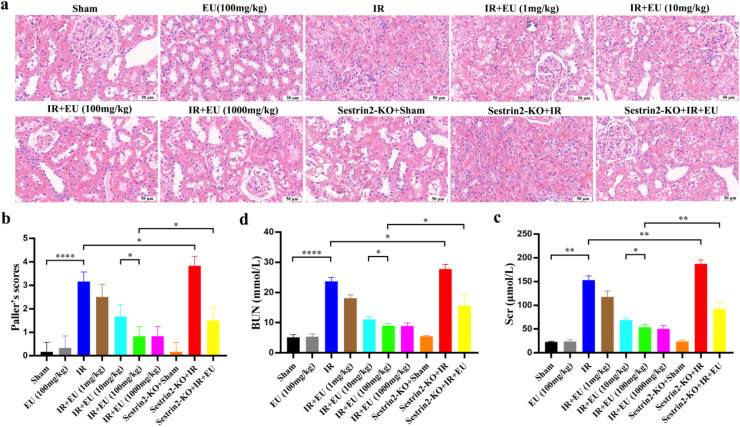
EU protects against RIRI in a dose-dependent manner. Mice were given a sham operation or IR with or without administration of EU (1, 10, 100 or 1000 mg/kg/day). (a) Representative images of HE-staining in the kidney sections (scale bar 50 μm); (b) The paller's scores of kidney injury; (c‒d) the serum concentrations of BUN and Scr of rats. * *p* < 0.05; ** *p* < 0.01; *** *p* < 0.001; **** *p* < 0.0001.

To elucidate the influence of EU on RIRI, mice received varying oral doses of EU (1, 10, 100, and 1000 mg/kg/day) for seven days before surgery. Post-surgery, renal function was evaluated. H&E staining and renal injury scoring revealed a dose-dependent reduction in renal tubular injuries induced by IR. Notably, significant mitigation occurred at doses of 100 mg/kg (*p* < 0.0001) and 1000 mg/kg (*p* < 0.0001; Fig. 1a‒b). Given comparable renal protection between 100 and 1000 mg/kg EU pretreatment (*p* > 0.999; Fig. 1a‒b), 100 mg/kg EU was selected for subsequent experiments. Additionally, EU pretreatment significantly decreased Scr and BUN levels compared to the IR group (Fig. 1c‒d), warranting a comprehensive exploration of the molecular mechanisms underlying EU's therapeutic effects on RIRI.

### *Contribution of Sestrin2 to EU's protective effect against RIRI*

**Fig. 2:**
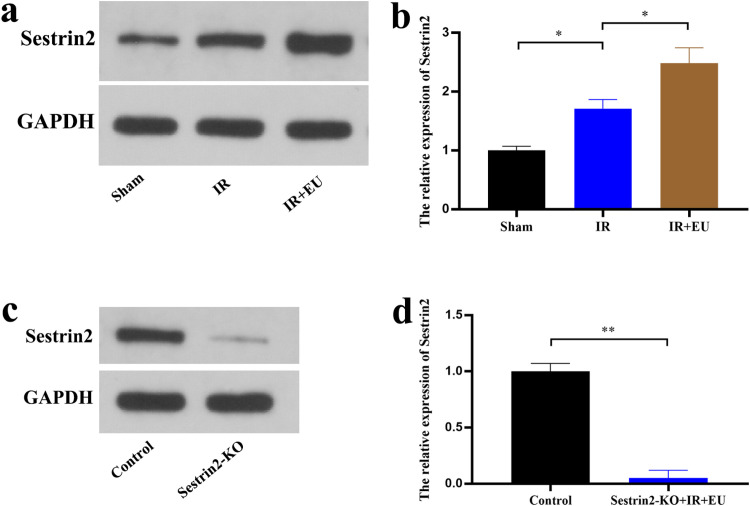
Sestrin2 is involved in the protective effect of EU against RIRI. (a‒b) Western blots were used to detect Sestrin2 expression during RIRI; (c‒d) Western blots were used to detect Sestrin2 expression in Sestrin2-KO mice. * *p* < 0.05; ** *p* < 0.01; *** *p* < 0.001; **** *p* < 0.0001.

Sestrin2, known for its role in regulating oxidative stress, exhibited increased expression after IR (*p* = 0.0465), further enhanced by EU treatment (*p* = 0.0435; Fig. 2a‒b). Utilizing Sestrin2 knockout (Sestrin2-KO) mice, the authors investigated the involvement of Sestrin2 in EU's protective effects against RIRI (Fig. 2c‒d). H&E staining and renal injury scoring unveiled exacerbated IR-induced renal tubular injury in Sestrin2-KO (*p* = 0.0179) and a reversal of EU's nephroprotective effects (*p* = 0.0379; Fig. 1a‒b). Furthermore, Sestrin2-KO elevated Scr and BUN concentrations during RIRI (*p* = 0.0266; *p* = 0.0081; Fig. 1c‒d).

### *EU's anti-oxidative stress ability mediated by Sestrin2*

**Fig. 3:**
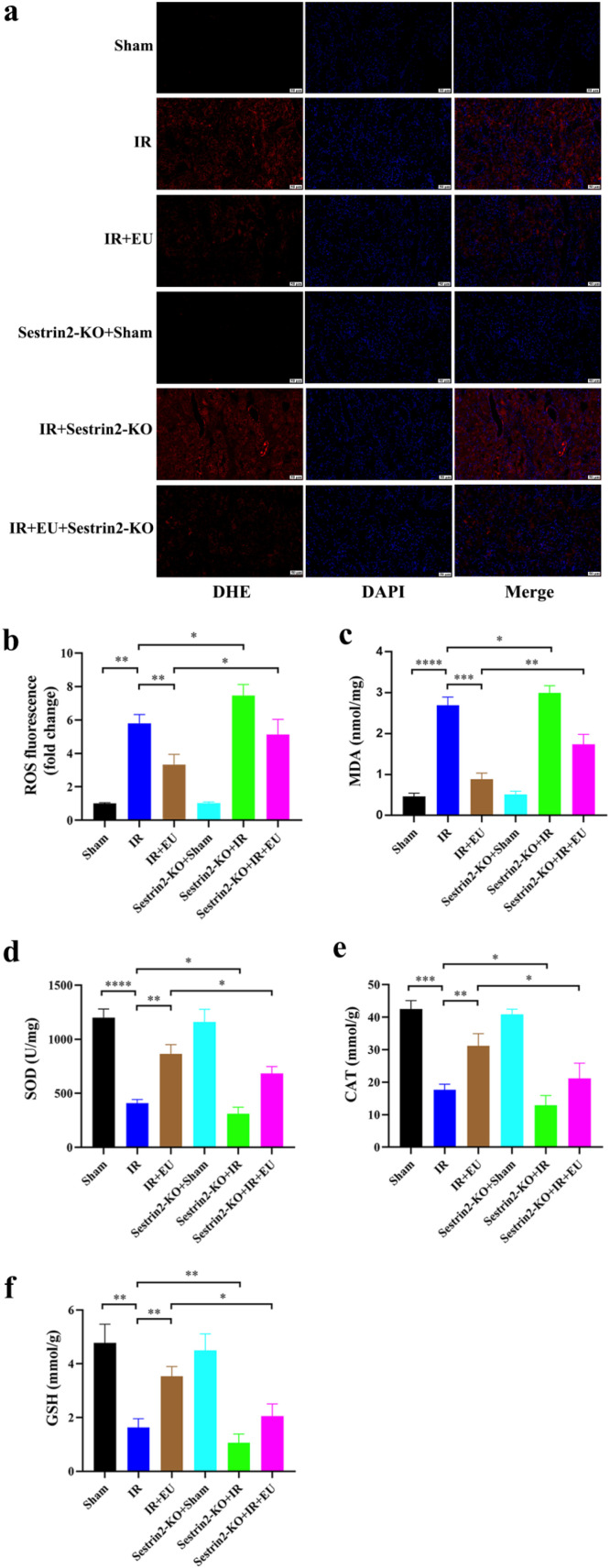
EU exerts anti-oxidative stress ability by regulating Sestrin2. (a‒b) Representative micrographs of kidney sections stained with DHE and semi-quantification analysis of fluorescence intensity indicated EU pretreatment reduced the ROS levels subjected to RIRI (scale bar 50 μm); (c‒f) the level of MDA, SOD, CAT and GSH in the kidney. * *p* < 0.05; ** *p* < 0.01; *** *p* < 0.001; **** *p* < 0.0001.

Exploring the impact of EU pretreatment on ROS levels in IR-induced kidneys, the present findings demonstrated a significant reduction in ROS and MDA levels, coupled with increased SOD, CAT, and GSH activities (*p* = 0.0061; *p* = 0.0002; *p* = 0.0029; *p* = 0.0048; *p* = 0.0026; Fig. 3a‒f). Intriguingly, Sestrin2 knockout (Sestrin2-KO) reversed EU's antioxidative effects, elevating ROS and MDA levels while diminishing SOD, CAT, and GSH content in IR-affected kidney tissues (*p* = 0.0458; *p* = 0.0066; *p* = 0.044; *p* = 0.0453; *p* = 0.0119; Fig. 3a‒f).

### *Sestrin2 plays a role in the protective effect of EU against HR-induced oxidative stress*

**Fig. 4:**
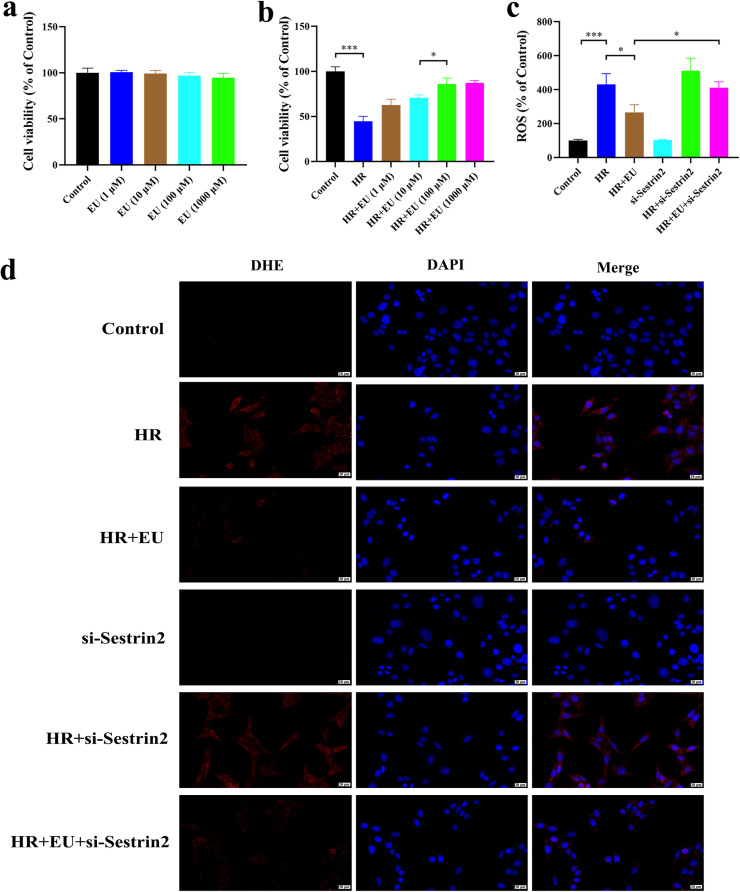
Sestrin2 is involved in the protective effect of EU on oxidative stress against HRI. (a) EU had no obvious toxic effect on TCMK-1 cells viability; (b) 1‒1000 μM EU has an obvious renal protective effect; (c‒d) representative images and statistical analysis of DHE staining indicated that EU pretreatment reduced ROS content in HR-induced TCMK-1 cells (scale bar 20 μm). * *p* < 0.05; *** *p* < 0.001.

**Fig. 5:**
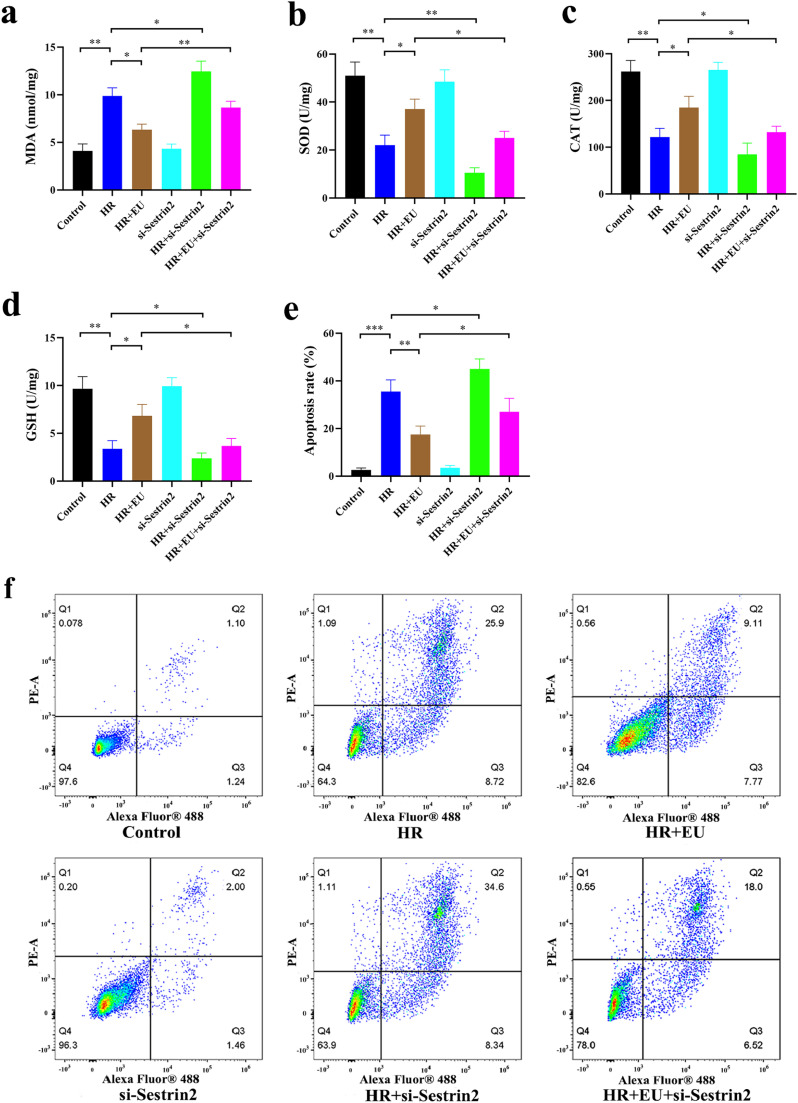
Sestrin2 is involved in the protective effect of EU on oxidative stress against HRI. (a‒d) The level of MDA, SOD, CAT and GSH in HR-induced TCMK-1 cells; (e‒f) Representative micrographs and the statistical results of the flow cytometry. * *p* < 0.05; ** *p* < 0.01; *** *p* < 0.001.

Evaluation of EU's impact on Hypoxia-Reoxygenation Injury (HRI) and Sestrin2′s role revealed no significant toxicity of EU on mouse renal tubular epithelial cell line TCMK-1 viability after 24 h (Fig. 4a). Subsequent experiments showcased EU's reduction of HR-induced cell death, particularly at 100 μM concentration (*p* = 0.0220; Fig. 4b). DHE staining (*p* = 0.0231; Fig. 4c‒d), MDA, SOD, CAT, and GSH assays (*p* = 0.0416; *p* = 0.0359; *p* = 0.0458; *p* = 0.0451; Fig. 5a‒d) demonstrated EU's ability to decrease HR-induced ROS levels and apoptotic cell numbers (*p* = 0.0021; Fig. 5e‒f). Importantly, Sestrin2-KO reversed EU's protective effects, promoting oxidative stress and cell viability inhibition (*p* = 0.0121; *p* = 0.0021; *p* = 0.0311; *p* = 0.0461; *p* = 0.0453; *p* = 0.0351; Fig. 4c‒d, Fig. 5a‒f).

### *Sestrin2 attenuates ERS in RIRI*

**Fig. 6:**
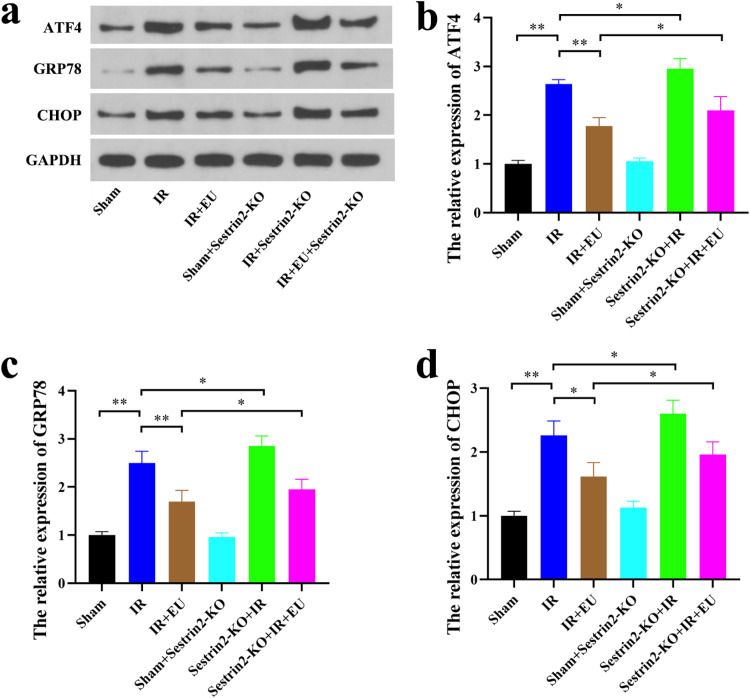
Sestrin2 attenuates RIRI-related ER. (a‒d) Western blots were used to detect the expression of ATF4, GRP78 and CHOP during RIRI. * *p* < 0.05; ** *p* < 0.01; *** *p* < 0.001; **** *p* < 0.0001.

The intricate relationship between ERS and intracellular redox status prompted an exploration into whether Sestrin2′s antioxidant prowess contributes to ERS attenuation during RIRI. Western blot analysis revealed that in the Sham and Sham+Sestrin2-KO groups, the expression levels of ATF4, GRP78, and CHOP remained comparable and unaffected by Sestrin2 expression variations (Fig. 5a‒d). However, post-IR, a substantial increase in GRP78 expression was evident (*p* = 0.0067), particularly pronounced in the Sestrin2-KO group (*p* = 0.0421; Fig. 5a‒d). Moreover, ATF4 and CHOP expressions were significantly upregulated after IR (*p* = 0.0023; *p* = 0.0078), with Sestrin2-KO exacerbating this effect (*p* = 0.0467; *p* = 0.0453; Fig. 5a‒d). Importantly, EU pretreatment significantly curtailed ATF4, GRP78, and CHOP expression in ischemia-reperfusion-injured kidneys (*p* = 0.0067; *p* = 0.0089; *p* = 0.0121). However, the inhibitory effect of EU on these genes was nullified by Sestrin2-KO (*p* = 0.0457; *p* = 0.0425; *p* = 0.0323; Fig. 6a‒d). In summary, EU pretreatment demonstrated the inhibition of ERS induced by RIRI, while the suppression of Sestrin2 hindered EU's anti-ERS efficacy.

## Discussion

RIRI remains a major cause of AKI, especially following surgical interventions such as partial nephrectomy and kidney transplantation, contributing significantly to the morbidity and mortality associated with AKI.^12^ The quest for effective preventive and therapeutic strategies against RIRI highlights its clinical importance. Extensive research has delineated the complex pathophysiological mechanisms of RIRI, which encompass the dysregulation of genes, various regulatory factors, and changes in renal microcirculation.^13^^,^^14^ During IR, renal cells experience a range of pathological responses, including increased synthesis of oxygen free radicals, depletion of antioxidants, reduced ATP production, and electrolyte disturbances, all of which contribute to oxidative stress, inflammation, and apoptosis. The accumulation of ROS and the reduction in antioxidant enzyme activity are key drivers of RIRI-induced renal damage.^15^ Notably, the aberrant activation of the xanthine oxidase pathway during IR serves as a significant source of ROS, exacerbating oxidative damage to lipids, proteins, and DNA, leading to structural and functional cellular impairments.^16^

Antioxidant enzymes, such as SOD, GSH, and CAT, play crucial roles in scavenging ROS within kidney cells, targeting specific ROS.^17^ MDA, a product of lipid peroxidation, is a reliable biomarker for assessing tissue ROS levels, reflecting the susceptibility of lipids to oxidative damage.^18^ To maintain physiological homeostasis, renal cells balance oxidative stress through the regulated generation and elimination of ROS.^19^

The Sestrin protein family, which is highly conserved and induced by various stress responses, including oxidative stress, plays a critical role in mitigating RIRI.^20^ Comprising three members (Sestrin1–3), Sestrins are generally expressed in human tissues, with Sestrin2, also known as hypoxia-inducible gene 95, holding particular significance.^21^ Sestrin2 is distributed across various human organs, including the kidneys, lungs, white blood cells, liver, gastrointestinal tract, and the brain.^22^ Sestrin2 has been shown to have substantial cytoprotective effects under different stress conditions and can be upregulated by diverse stimuli, such as DNA damage, oxidative stress, ischemia, hypoxia, Endoplasmic Reticulum Stress (ERS), and alterations in energy metabolism.^23^^,^^24^ Moreover, Sestrin2 exhibits a range of biological functions, encompassing the inhibition of DNA damage, oxidative stress, ERS, and ferroptosis.^25^ The data further confirmed that elevated oxidative stress levels associated with RIRI induce Sestrin2 expression, and the upregulation of Sestrin2 induced by EU significantly mitigates RIRI. These findings emphasize the potential of Sestrin2 upregulation as a therapeutic strategy for RIRI.

Given that Sestrin2 is regulated by multiple transcription factors, including p53, Hypoxia-Inducible Factor 1-alpha (HIF-1α), and Nuclear Factor erythroid 2-related factor 2 (Nrf2), it is plausible that these factors may play a role in the EU-mediated protective effects.^26^^,^^27^ It is well established that Nrf2 activation leads to the induction of several antioxidant enzymes, including Heme Oxygenase-1 (HO-1), which combats oxidative stress.^27^^,^^28^ Moreover, the Nicotinamide Adenine Dinucleotide Phosphate (NADPH) oxidase pathway, which is involved in the production of ROS, is also affected by Nrf2 signaling.^29^^,^^30^ Thus, the interplay between Nrf2, HO-1, and NADPH oxidase in the context of RIRI, along with the modulation of Sestrin2, warrants further investigation.

EU, a primary component of clove oil, is recognized for its potent natural antioxidant properties.^31^ The present study aligns with previous findings emphasizing the antioxidant properties of EU compounds in cloves.^6^ Magdalena et al.^32^ have confirmed that EU in cloves is an important class of antioxidants. In addition, Chaieb et al.^33^ demonstrated that EU and gallic acid in cloves exhibited significant antioxidant capacity. EU has a strong scavenging effect on·OH and O_2_^−^.^34^ The remarkable biological properties of the EU have found application in the treatment of various diseases.^35^, ^36^, ^37^, ^38^

This study demonstrates that EU treatment results in a significant reduction in RIRI and an increase in Sestrin2 expression. Notably, the renoprotective effects of EU are diminished when Sestrin2 is knocked down, indicating that EU exerts its protective effects, at least in part, through the upregulation of Sestrin2. However, it is important to clarify that the western blotting analysis evaluates protein expression rather than gene expression. Changes in gene expression do not always correlate with corresponding changes in protein levels or downstream functional outcomes. Therefore, to better elucidate the mechanisms by which EU ameliorates RIRI, the authors should consider the involvement of downstream components of the apoptotic pathway.

CHOP is a key mediator of ERS-induced apoptosis, and it promotes cell death by suppressing the anti-apoptotic Bcl-2 and activating pro-apoptotic Bim and Bax proteins.^39^ The present result suggested that the EU inhibits ERS during RIRI, potentially via the regulation of Sestrin2. To provide a more comprehensive understanding, future studies could investigate the impact of EU on the expression of CHOP, Bcl-2, Bim, and Bax, as well as their functional interactions, to fully characterize the anti-apoptotic mechanisms underlying the renoprotective effects of EU.

In conclusion, the EU effectively ameliorates oxidative damage and ERS caused by RIRI, highlighting the intricate relationship between oxidative stress, Sestrin2, and EU in the context of RIRI. Further elucidation of the molecular mechanisms, including the evaluation of apoptosis-related proteins, could pave the way for novel therapeutic approaches to combat RIRI.

## Conclusion

The present investigation affirms the efficacy of EU in alleviating RIRI and HRI in a murine model. The renoprotective attributes of EU are associated with the suppression of oxidative stress, ERS, and the concurrent upregulation of Sestrin2.

## Patient consent for publication

Not applicable.

## Statement of ethics

All animal studies adhered to the ARRIVE guidelines and were approved by the Ethics Committee of Qingdao Chengyang People's Hospital (Approval n° Q2021–04–015).

## Data availability statement

All data generated or analyzed during this study are included in this article. Further inquiries can be directed to the corresponding author.

## Funding

The authors did not receive any funding.

## Declaration of competing interest

The authors declare no conflicts of interest.
